# Supplementary material for: Overcoming the threat of anti-bias interventions: Combining self-report and psychophysiological measures to capture the process of change
Source: PLoS One. 2025 Jan 13;20(1):e0314813. doi: 10.1371/journal.pone.0314813 (PMC11730427; doi:10.1371/journal.pone.0314813)
Supplement: S1 Appendix — (DOCX) [file pone.0314813.s001.docx]

**S1. Appendix. Teacher Evaluation Survey**

Online questionnaire before the intervention

Prior to taking part in the lab experiment, participants were invited to evaluate two lecturers and two workgroup teachers of courses they had recently attended (via the online survey platform Qualtrics). Participants first indicated how desirable it is *in general* for a teacher to show certain teaching behaviours and possess certain characteristics, and then evaluated the four teachers on these teaching behaviours and characteristics. They also provided an overall assessment of each teacher and course, as well as information about the gender of the teacher, the (estimated) seniority, the (estimated) teaching experience and the estimated/obtained grade for the course.

**Desirability.** Participants were shown eight teaching behaviours and characteristics related to effective teaching (e.g., “*Cares about and understands their students, and has their students’ best interests at heart.*”, “*Is decisive, disciplined and confident in leading their class and giving presentations.*”, “*Is honest and trustworthy to students, works under a set of morals and ethics, and is genuine*.”). They were asked to rate how desirable they thought each of these behaviours and characteristics are on a scale from 1 (*not desirable*) to 5 (*extremely desirable*).

**Details course type and teacher.** Participants were asked several questions about the lecture and workgroup teachers and courses they were evaluating. Information was requested about the gender of the teacher (reported in S1 Fig), whether the teacher was a junior staff member or a senior staff member, how much teaching experience they thought the teacher had (i.e., *no experience, a little experience, a moderate amount of experience, a lot of experience, a great deal of experience*), and in which quarter of the past academic year they took the course. They were asked these questions four times, once for each lecture teacher and once for each workgroup teacher.

**Student evaluation of teachers.** Participants were asked to what extent the eight teaching behaviours and characteristics described the teacher on a scale from 1 (*does not describe him/her*) to 5 (*describes him/her extremely well*). They also gave the teacher a star-review (between one and five stars) to indicate their overall assessment of the teacher. Next, participants were asked to rate three statements about their overall experience with the lectures or workgroups *(*i.e., “*The lectures were interesting and inspiring.*”, “*The lectures were useful and educational.*”, “*It was easy to remain attentive during the lectures*.”) on a 7-point Likert scale (1 = *strongly disagree –* 7 = *strongly agree*), and two ratings about how easy the lectures/workgroups and examinations were on a 7-point Likert scale (1 = *extremely difficult –* 7 = *extremely easy*). They were then asked to give an overall assessment of the course with the same five-star rating system as for their overall assessment of their teacher. Finally, they were asked if they had already gotten a grade for the course and if so, what the grade was, or if not, what grade they expected to get. Participants were asked to answer these questions four times, once for each lecturer and once for each workgroup teacher.

Results

In total 239 male teachers and 165 female teachers were evaluated. Most participants (43.6%) evaluated two male and two female teachers, but there were also many participants (34.7%) that evaluated three male teachers and one female teacher, see also Fig S1. The participants who only evaluated teachers of the same gender (N=16) were excluded when testing for gender bias in teacher evaluations.

**Fig S1***.* **Distribution of the gender composition of evaluated teachers per evaluating students.**


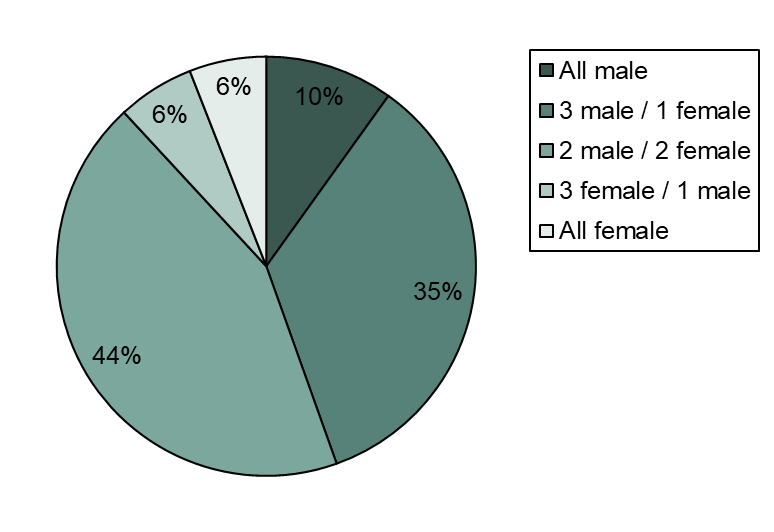
Overall, the scores for lecture and workgroup teachers were negatively skewed with many high scores in the distribution (L1= -. 54; L2= - .42; Wg1= - .36; Wg2= - .63). The Shapiro-Wilk's tests that were applied to check the assumption of normality for each lecture and workgroup teacher’s overall assessment were all highly significant (all p's < .001), meaning that the overall teacher scores significantly deviated from a normal distribution. Additionally, the pp-plots also showed a non-normal pattern.

**Student evaluation of teachers.** We first verified whether it was necessary to consider covariates when checking for gender bias in teacher evaluations. Overall, more senior teachers (61.8%) were evaluated than junior teachers (38.2%), especially within the first evaluated lecture (78.8%, *X^2^* = 38.11, *p* < .001, *phi =* .34). Further, as the evaluated senior teachers were often male (63.3%, *X^2^* = 5.22, *p* = .022, *phi* = -.12), seniority was included as a covariate in subsequent analyses. The gender composition among the junior teachers was equal (50.8% male and 49.2% female).

When seniority and course type were taken into account, no differences were found in the assessment of male and female teachers on the assessed teaching behaviours and characteristics (*F*’s(1,337) ≤ 3.80, *p*’s ≥ 0.052), overall assessment of the teacher (*F*(1,337) = .39, *p* = .534), and overall satisfaction with the lecture / workgroup (*F*(1,338) =1.56, *p* = .212). Thus, on the assessed student evaluation indicators, the participants showed no gender bias in the evaluation of teachers to the disadvantage of female teachers.
