# Supplementary material for: Overcoming the threat of anti-bias interventions: Combining self-report and psychophysiological measures to capture the process of change
Source: PLoS One. 2025 Jan 13;20(1):e0314813. doi: 10.1371/journal.pone.0314813 (PMC11730427; doi:10.1371/journal.pone.0314813)
Supplement: S2 Appendix — (DOCX) [file pone.0314813.s002.docx]

**S2. Appendix. Additional Measures Lab Session**

Self-report measures assessed after the intervention

In addition to the self-report measures reported in the main manuscript, the measures described hereafter were also assessed after the entire intervention, see also S1 and S2 Tables. Therefore, all dependent variables described below were examined with a 2 (Self-implied: Yes vs. No) x 2 (Frame: Promotion vs. Prevention) ANOVA. All items were rated on a 7-point Likert scale (1 = *strongly disagree* – 7 = *strongly agree*), unless otherwise indicated.

**Table S1. Correlations Table for Self-report measures.**

|  | 1 | 2 | 3 | 4 | 5 | *6* | *7* | *8* | *9* | 10 | 11 | 12 | 13 | 14 |
| --- | --- | --- | --- | --- | --- | --- | --- | --- | --- | --- | --- | --- | --- | --- |
| 1. Opportunities vs. Risks Scale | . |  |  |  |  |  |  |  |  |  |  |  |  |  |
| 2. Acceptance of Feedback Component 1 | 0.070 | . |  |  |  |  |  |  |  |  |  |  |  |  |
| 3. Acceptance of Feedback Component 2 | 0.385^**^ | -0.020 | . |  |  |  |  |  |  |  |  |  |  |  |
| 4. Acceptance of Feedback Component 3 | -0.080 | 0.263 | 0.010 | . |  |  |  |  |  |  |  |  |  |  |
| 5. Future Intentions to regulate bias | 0.067 | 0.143 | -0.035 | -0.441^**^ | . |  |  |  |  |  |  |  |  |  |
| 6. Bias Persist | 0.296^**^ | 0.093 | -0.176 | 0.009 | 0.066 | . |  |  |  |  |  |  |  |  |
| 7. Bias Indicative | 0.185 | -0.265 | 0.259 | -0.243 | -0.078 | .268^**^ | . |  |  |  |  |  |  |  |
| 8. Bias Predictive | 0.230^*^ | -0.126 | 0.144 | -0.325^*^ | 0.163 | 0.133 | 0.321^**^ | . |  |  |  |  |  |  |
| 9. Bias Not Predictive | 0.068 | -0.041 | 0.006 | -0.248 | 0.195 | 0.232^*^ | 0.133 | 0.201^*^ | . |  |  |  |  |  |
| 10. Threat Appraisal | 0.319^**^ | -0.137 | 0.218 | -0.105 | 0.027 | 0.087 | 0.163 | 0.277^**^ | -0.039 | . |  |  |  |  |
| 11. Perceived Coping Ability Speech 1 | -0.097 | 0.001 | 0.053 | -0.155 | 0.201^*^ | -0.092 | 0.026 | -0.036 | 0.121 | -0.308^**^ | . |  |  |  |
| 12. Perceived Coping Ability Speech 2 | -0.416^**^ | 0.149 | -0.202 | 0.372^**^ | -.210^*^ | -0.264^**^ | -0.052 | -.196^*^ | -0.227^*^ | -0.239^*^ | -0.050 | . |  |  |
| 13. Positive and Negative reactions | 0.118 | -0.431^**^ | 0.213 | 0.112 | -0.462^**^ | 0.085 | 0.348^**^ | 0.039 | -0.043 | 0.312^**^ | -0.121 | 0.070 | . |  |
| 14. Belief in Gender Discrimination | -0.001 | 0.306^*^ | -0.072 | -0.051 | 0.321^**^ | -0.028 | -.326^**^ | -0.082 | 0.080 | -0.163 | 0.131 | -0.082 | -0.616^**^ | . |

*Note.* ** Correlation is significant at the 0.01 level (2-tailed).

* Correlation is significant at the 0.05 level (2-tailed).­­­

^a^ Initially, these three items were considered as belonging to a single scale: Perceived stability of Behavior. However, due to the low reliability and seemingly different constructs, it was decided to analyze the items as separate dependent variables. Item 1: *I think that the bias in student evaluations of teachers will persist and remain stable over time.*

^b^ Item: *I think the bias in the evaluations of teachers is indicative of my true nature.*

^c^ Item: *I think the bias in the evaluations of teachers is predictive of my future behavior.*

^d^ Item: *I think the bias in the evaluations of teachers does not predict the behavior of students over time and is subject to change (reversed).*

**Table S2. Descriptive Statistics: Means (M) and Standard Deviations (SD) for Self-Report Variables.**

|  | | **Self-Implied**  **(N=50)** | **Self Not-Implied**  **(N=51)** | **Prevention**  **(N=48)** | **Promotion**  **(N=53)** | **Total**  **(N=101)** |  |
| --- | --- | --- | --- | --- | --- | --- | --- |
|  |  |  |  |  |  |  |  |
| **Opportunities vs. Risks Scale** | M | 3.88 | 3.90 | 3.78 | 4.00 | 3.89 |  |
|  | SD | 0.84 | 0.95 | 0.84 | 0.93 | 0.89 |  |
| **Acceptance of Feedback** | |  |  |  |  |  |  |
| Component 1 | M | 4.55 |  | 4.55 | 4.56 | 4.55 |  |
|  | SD | 1.67 |  | 1.81 | 1.59 | 1.67 |  |
| Component 2 | M | 3.47 |  | 3.52 | 3.43 | 3.47 |  |
|  | SD | 1.13 |  | 1.11 | 1.17 | 1.13 |  |
| Component 3 | M | 3.61 |  | 3.71 | 3.54 | 3.61 |  |
|  | SD | 1.46 |  | 1.43 | 1.50 | 1.46 |  |
| **Future Intentions to regulate bias** | M | 4.37 | 4.51 | 4.49 | 4.40 | 4.44 |  |
|  | SD | 1.17 | 1.20 | 1.23 | 1.14 | 1.18 |  |
| **Perceived Stability of Behavior** | |  |  |  |  |  |  |
| Bias Persist ^a^ | M | 3.80 | 4.16 | 3.90 | 4.06 | 3.98 |  |
|  | SD | 1.29 | 1.48 | 1.42 | 1.38 | 1.39 |  |
| Bias Indicative ^b^ | M | 2.98 | 2.92 | 2.88 | 3.02 | 2.95 |  |
|  | SD | 1.41 | 1.47 | 1.51 | 1.37 | 1.43 |  |
| Bias Predictive ^c^ | M | 2.78 | 3.33 | 2.98 | 3.11 | 3.05 |  |
|  | SD | 1.37 | 1.74 | 1.44 | 1.72 | 1.58 |  |
| Bias Not Predictive ^d^ | M | 3.16 | 3.51 | 3.31 | 3.34 | 3.33 |  |
|  | SD | 1.34 | 1.38 | 1.59 | 1.14 | 1.36 |  |
| **Threat Appraisal** | M | 3.01 | 2.97 | 2.90 | 3.07 | 2.99 |  |
|  | SD | 1.25 | 1.22 | 1.06 | 1.37 | 1.23 |  |
| **Perceived Coping Ability** | |  |  |  |  |  |  |
| Speech 1 | M | 3.41 | 3.75 | 3.49 | 3.66 | 3.58 |  |
|  | SD | 1.13 | 1.29 | 1.05 | 1.36 | 1.22 |  |
| Speech 2 | M | 3.49 | 3.73 | 3.85 | 3.40 | 3.61 |  |
|  | SD | 1.20 | 1.47 | 1.38 | 1.28 | 1.34 |  |
| **Positive and Negative Reactions** | M | 2.01 | 2.13 | 2.05 | 2.08 | 2.07 |  |
|  | SD | 0.80 | 0.74 | 0.74 | 0.81 | 0.77 |  |
| **Belief in Gender Discrimination** | M | 5.49 | 5.15 | 5,132 | 5,491 | 5.32 |  |
|  | SD | 0.84 | 1.02 | 1.01 | 0.86 | 0.95 |  |

*Note.* Initially, the four items Bias Persist, Bias Indicative, Bias Predictive and Bias Not Predictive were considered as belonging to a single scale: Perceived stability of Behavior. However, due to the low reliability and seemingly different constructs, it was decided to analyze the items as separate dependent variables:

^a^ Item 1: *I think that the bias in student evaluations of teachers will persist and remain stable over time.*

^b^ Item: *I think the bias in the evaluations of teachers is indicative of my true nature.*

^c^ Item: *I think the bias in the evaluations of teachers is predictive of my future behavior.*

^d^ Item: *I think the bias in the evaluations of teachers does not predict the behavior of students over time and is subject to change (reversed).*

**Perceived stability of behaviour.** The perceived stability of the teacher evaluation bias was measured with four items adapted from Pagliaro, Ellemers, Barreto and Cesare (2016) to examine the extent to which participants believed they could influence the bias and thought it subject to change *(*e.g., “*I think that the bias in student evaluations of teachers will persist and remain stable over time.*”, “*I think the bias in the evaluations of teachers is indicative of my true nature.*”*).* Reliability for the four-item scale was low (*α* = .52) and could not be improved by removing any of the items. Therefore, the four items were analysed as separate dependent variables in a MANOVA. We found no significant main effect of the self-implied manipulation, *F*(4,93) = 1.53, *p* = .20, framing manipulation, *F*(4,93) = 0.17, *p* = .96, *η*^2^ = .06 nor an interaction effect between self-implication and framing, *F*(4,93) = 0.36, *p* = .83, *η*^2^ = .01. Thus, there were no differences between participants in the belief that the bias could be influenced and was subject to change.

**Threat appraisal*.*** Four items adapted from Skinner and Brewer (2002) were used to examine participants’ tendency to focus on possible harm to one’s self-esteem and social identity (e.g., “*I worry about what other people will think of students when they hear about the bias in the evaluation of teachers.*”, “*I am concerned that others will blame me once they hear about the bias in the evaluation of teachers.*”)*.* Reliability for the four-item threat appraisal scale was acceptable (*α* = .73). There was no significant main effect of self-implied, *F*(1,97) = 0.01, *p* = .93, nor promotion-prevention focus, *F*(1,97) = 0.43, *p* = .51, nor the interaction, *F*(1,97) = 0.001, *p* = .98.

**Positive and negative reactions to teacher evaluation bias**. Nine items, adapted from Parker et al. (2016), were used to examine participants’ thoughts and opinions in reaction to the teaching evaluation bias (e.g., “*I think it is good that action is taken within academia to decrease the difference in the way female and male teachers are treated.*”, “*The difference in ratings between male and female teachers shows that women simply do not have the talent necessary to be great teachers.*”). Reliability for the nine-item positive and negative reactions to teacher evaluation bias scale was acceptable (*α* =.78). Results showed no significant main effect of self-implication, *F*(1,97) = 0.80, *p* = .37, framing, *F*(1,97) = 0.09, *p* = .76, nor an interaction effect between self-implication and framing, *F*(1,97) = 2.54, *p* = .12. In general, participants reported relatively low negative responses to the teacher evaluation bias (all condition *M’s* ≤ 2.23).

**Attention checks.**At the end of the study several items were included to check whether participants had paid attention during the experiment. They were asked what teacher evaluations are used for (open-ended), whether they got to see the results from the online study they had done (yes or no), and whether they had seen a video about the university’s ideals and ambition or about the university’s obligations and responsibility. None of the participants failed all three attention checks (one of our exclusion criteria; 94,1% of participants responded correctly for at least one of two control questions).
