# Supplementary material for: Overcoming the threat of anti-bias interventions: Combining self-report and psychophysiological measures to capture the process of change
Source: PLoS One. 2025 Jan 13;20(1):e0314813. doi: 10.1371/journal.pone.0314813 (PMC11730427; doi:10.1371/journal.pone.0314813)
Supplement: S3 Appendix — (DOCX) [file pone.0314813.s003.docx]

**S3. Appendix. Supporting Information. Speech Data**

Content analyses of both speeches

In exploring what participants talked about during Speech 1 and 2, responses were coded based on predefined response-categories relevant to the purpose of the research.

Regarding Speech 1, we defined the following response-categories: the presence or absence of quotes regarding *the self* (i.e., whether participants referred to themselves, 0/1); quotes regarding *other people* (i.e., referring to the group or society as a whole, 0/1); the notion of *prescriptive stereotypes* (i.e., prescriptive expectations students have about males and females and how this affects their teaching style, 0/1) or *descriptive stereotypes* (i.e., whether male/female teachers behave differently, 0/1); the number of *social arguments* (e.g., the society is to blame, comments about representation that teachers have always been male, stereotypes) and *biological arguments* (e.g., the difference/preference is evolutionary ingrained); the *presence or absence of quotes that indicated surprise*, 0/1 (out of the five basic emotions, surprise seemed the most relevant to examine); and the *number of* times that participants used *words that indicated hesitance* (i.e., maybe, perhaps, I am not sure, I guess, or whatever, like, could be, I think, kind of, sort of).

Regarding Speech 2, we defined the following response-categories: the presence or absence of placing *responsibility on themselves* (0/1) or *on others* (0/1); amount of *do’s and don'ts* (i.e., distinguish behaviours aimed at achieving fair and just evaluations vs. behaviours aimed at avoiding unfair evaluations of teachers); the *presence or absence of including students* (i.e., ideas or measures that include students in them such as awareness training, 0/1) or *excluding students* (i.e., ideas or measures that exclude students such as changing the system, 0/1); the notion of *positive consequences* (i.e., positive results that reduce bias in teaching evaluations, 0/1) or *negative consequences* (i.e., reversed sexism, 0/1), and the number of *hesitance words* that were mentioned.

Below we describe the general characteristics of both speeches, as there were no apparent differences between conditions. When significant differences did arise, this is reported.

Results

**Speech 1.** In terms of content, most participants deflected the blame when talking about where the teaching bias comes from, by mostly mentioning other people (59.4%) rather than themselves (38.6%). Additionally, most arguments explained the difference in teaching evaluations by assuming that male and female teachers behave differently for social reasons, such as the way boys and girls are raised (86.1%) or referring to biological differences between men and women (such as their tone of voice) to explain more positive responses to male teachers (15.8%). Most participants in Speech 1 mentioned descriptive stereotypes (87.1%), describing how female and male teachers are and behave differently rather than using prescriptive stereotypes (17.8%) of expectations people have about males/females and how this should affect their teaching style.

Very few participants (8.9%) expressed surprise during their first speech (both in the self-implied and the not self-implied condition). There were no significant differences in the absence or presence of surprise between the self-implied and the not self-implied condition (*X^2^* = 0.101, *p* = 0.75). A high percentage of participants used hesitant words in their speeches (87,1%) during Speech 1, however this amount was higher in the self-implied (*M* = 3.42, *SD* = 2.73) than in the not self-implied condition (*M* = 2.57, *SD* = 2.22), 88% and 86.3% respectively, *t*(99) =1.72, *p* = 0.09, 95% CI [-0,13, 1,83], Hedges's g_s_ = 0.34].

**Speech 2.** Regarding the content of the second speech, in which participants were asked to reflect on what should be done to overcome gender bias in teacher evaluations, participants mainly assigned responsibility for achieving this to others (96%). A much smaller proportion referred to themselves as having a role in overcoming gender bias (22%). Further, participants used significantly more “do’s” (43%) than “don’ts” (18%) when considering ways to overcome gender bias. They were more likely to mention more “do’s” in the prevention condition (*M* = 1.65, *SD* = 1.21) rather than the promotion condition (*M* = 0.17, *SD* = 0.43, *t*(99) = -8.32, *p* < .001, 95% CI [-1.83, -1.12, Hedges's g_s_ = 1.65). While this may seem inconsistent with prior research showing that framing social equality between groups in terms of (moral) obligations compared to (moral) ideals, results in more don’ts (Does et al., 2010), our data suggest that as our participants were focusing on other people’s responsibilities, they did so in in terms of what these*other* people *should do*. Most participants suggested both including (89%) and excluding (74%) students from the ideas/measures for the reduction of bias. Additionally, participants mentioned more positive (48%) than negative consequences (20%) of promoting/preventing bias in teachers’ evaluations.

A similar percentage of hesitant words was used in both speeches (88% in Speech 2).

There were no significant differences in the amount of hesitant words used in the second speech between the self-implied (*M* = 3.22, *SD* = 2.60) and self not-implied conditions (*M* = 3.32, *SD* = 2.39), 90% and 86% (*t*(98) = -0.2, *p* = 0.84), nor between the amount of hesitance words used in the promotion (*M* = 3.62, *SD* = 2.34) and the prevention conditions (*M* = 2.90, *SD* = 2.60), 92.3% and 83.3% respectively, (*t*(98) = 1.46, *p* = 0.15).
